# Deep brain stimulation combined with morroniside promotes neural plasticity and motor functional recovery after ischemic stroke

**DOI:** 10.3389/fphar.2024.1457309

**Published:** 2024-12-04

**Authors:** Yanxi Chen, Zhidong Xu, Yifu Ma, Tingting Liu, Xin Tian, Zixin Zhu, Wenrong Zheng, Yufeng Wang, Ruifang Zheng, Jianguo Xing, Wen Wang, Fangling Sun

**Affiliations:** ^1^ Department of Experimental Animal Center, Xuanwu Hospital of Capital Medical University, Beijing Municipal Geriatric Medical Research Center, Beijing, China; ^2^ School of Chemical and Pharmaceutical Engineering, Hebei University of Science and Technology, Shijiazhuang, China; ^3^ Key Laboratory of Uighur Medicine of Xinjiang Uygur Autonomous Region, Xinjiang Institute of Materia Medica, Urumqi, China

**Keywords:** ischemic stroke, deep brain stimulation, morroniside, endogenous neurogenesis, motor function recovery

## Abstract

**Background and Objective:**

Until now, there has been an unmet need for treatments promoting chronic-phase post-stroke functional recovery. We previously found that morroniside promoted endogenous neurogenesis in ischemic stroke, but its therapeutic window was limited to the first 48 h. Here, we aimed to explore whether deep brain stimulation (DBS) combined with morroniside could enhance neurogenesis in rats subjected to focal ischemic stroke and contributes to functional recovery.

**Methods:**

Beginning 2 weeks after the endothelin-1-induced stroke, rats were administered DBS of lateral cerebellar nucleus consecutively for 14 days, followed by morroniside for 7 consecutive days post-stimulation. Behavioral tests were used for assessing motor function. Local field potentials were recorded to evaluate neuronal excitability. Nissl staining was used to assess infarct volume. Immunofluorescence staining and Western blotting were carried out to uncover the stroke recovery mechanisms of DBS combined with morroniside treatment.

**Results:**

The results showed that this combined treatment improved behavioral outcomes, enhanced cortical local field potentials, and diminished infarct volumes at 35 days post-stroke. Moreover, it notably amplified neurogenic responses post-stroke, evidenced by the proliferation of BrdU/SOX2 and BrdU/DCX in the subventricular zone, and their subsequent differentiation into BrdU/NeuN and BrdU/VgulT1 in the ischemic penumbra. Moreover, the combined treatment also elevated the amount of BrdU/Olig2 and the level of axonal sprouting-related proteins in the perilesional cortex.

**Conclusion:**

Our results demonstrated that the combined treatment extended the neurorestorative efficacy of morroniside, reduced infarct size, enhanced neuronal excitability and accelerated sensorimotor function recovery. This therapeutic approach may emerge as a potential clinical intervention for chronic ischemic stroke.

## 1 Introduction

Globally, ischemic injury can be regarded as the most commonly seen form of stroke, which usually results in chronic disability (Martin et al., 2024). At present, there are no reparative drugs for ischemic injury, leading to significant limitations in patients’ functional rehabilitation (Dhir et al., 2020). Ischemic injury can trigger endogenous repair processes. *De novo* neural stem cells (NSCs) and progenitor cells (NPCs) have been found in the subventricular zone (SVZ) of the adult mammalian brain, which migrated to ischemic penumbra in which they differentiated into mature neurons. However, spontaneous neurogenesis is insufficient for tissue repair and motor function restoration (Lindvall and Kokaia, 2015; Marques et al., 2019; Gozel and Gerstner, 2021). Consequently, enhancing ischemic-driven endogenous neurogenesis can probably be a promising approach to lower the burden of disease in patients. Our team is focused on finding effective strategies to enhance endogenous neurogenesis. We have demonstrated that morroniside (MNG), one of the most abundant iridoid glycosides isolated in *Cornus officinalis*, not only exerts the effect of neuroprotection (Wang et al., 2010), promotion of angiogenesis (Liu et al., 2016), and amelioration of microvascular dysfunction (Sun et al., 2014a), but more importantly, it could enhance endogenous neurogenesis and facilitate functional recovery following focal ischemic (Sun et al., 2014b). However, the optimal window for MNG to promote neurogenesis is within 48 h post-stroke. Beyond this window, its influence on neurogenesis notably diminishes. Additionally, MNG was found to be inactive in promoting neurogenesis in rats without ischemic injury. Consequently, we hypothesize that the effect of MNG depends on neurogenesis-related mechanisms induced by ischemia, and we are exploring effective methods to induce proliferation in NSCs, aiming to extend the neurogenesis window for MNG.

Deep brain stimulation (DBS), particularly within the lateral cerebellar nucleus (LCN), is regarded as a promising neurorestorative method. In several clinical studies, DBS contributes to stroke recovery with encouraging results. One clinical study involving 12 patients developing continuous (1–3 years) moderate-to-severe upper limb impairments underwent LCN DBS and found significant improvements in functional scores across all patients, along with high safety and tolerability (Baker et al., 2023). Additionally, LCN DBS demonstrated encouraging clinical outcomes in addressing post-stroke secondary tremors and spasticity (Teixeira et al., 2015; Cury et al., 2019; Brown et al., 2020; Pozzilli et al., 2023). Our previous study first discovered that 14 days of 30 Hz LCN DBS after brain ischemia enhances endogenous neurogenesis in the SVZ. This process leads to the differentiation of these cells into new neurons within the perilesional cortex, associated with functional recovery (Wu et al., 2020).

Capitalizing on DBS’s capacity to trigger neural stem cell proliferation, we speculate that LCN DBS may broaden the neurogenesis window of MNG. Here, we aim to explore whether combining DBS with MNG treatment could further enhance neurogenesis and accelerate functional recovery, providing an efficient treatment for chronic stroke. We built upon LCN DBS combined with MNG treatment performed in an ET-1-induced primary motor cortex ischemic stroke model. Firstly, we examined whether combination therapy reversed the impaired sensorimotor function and decreased neuronal activity, as well as the effect on infarct volume in the ET-1-induced stroke model. Next, we observed whether combination therapy could strengthen NPC proliferation, migration, and differentiation into mature neurons. We further observed the effect of combination therapy on neuroplasticity.

## 2 Results

### 2.1 Deep brain stimulation combined with morroniside treatment improved sensory−motor functions in stroke rats

**FIGURE 1:**
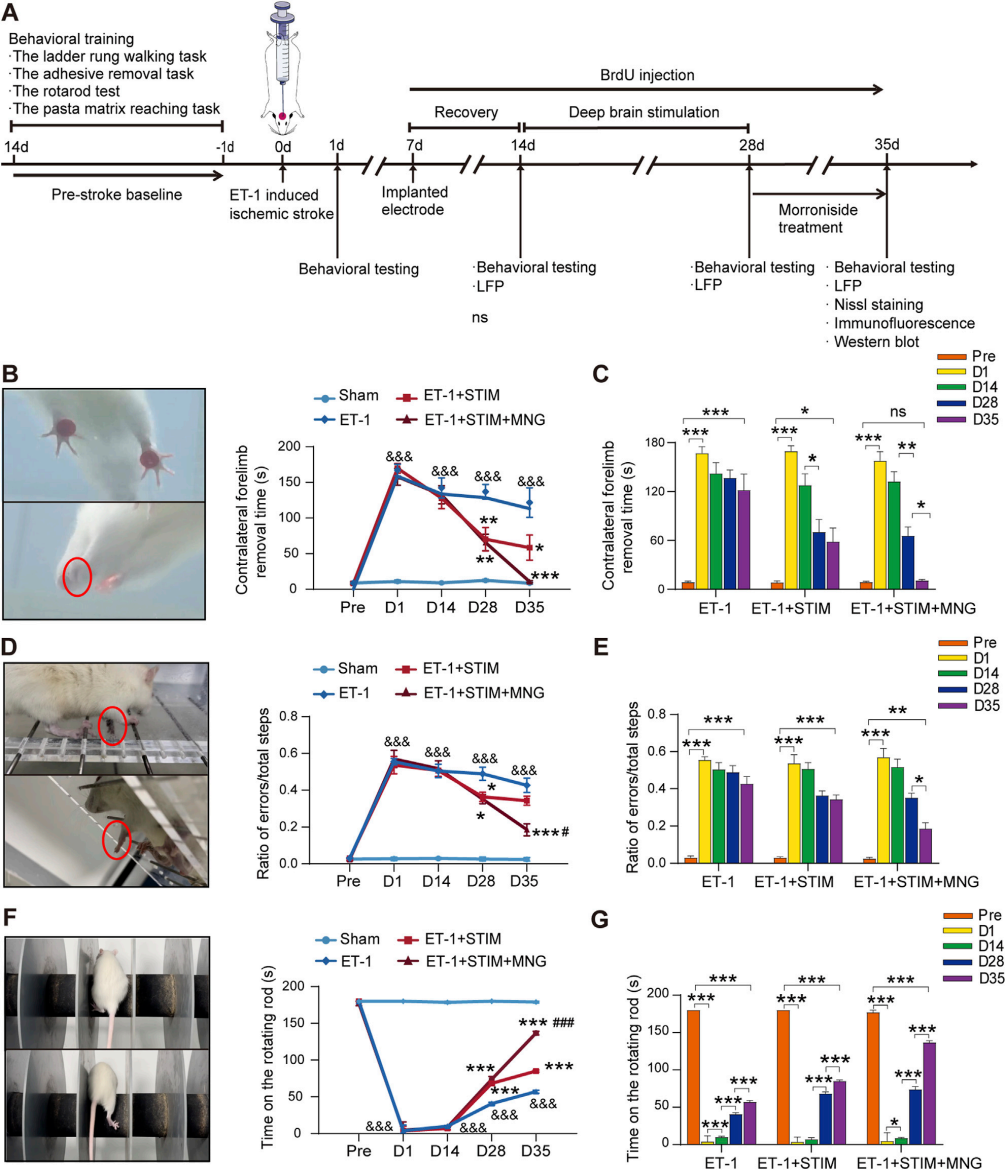
Deep brain stimulation combined with morroniside treatment improved sensory−motor function after stroke **(A)** The schedule and design of the experiment. **(B–G)** Quantification of the sensory−motor function test results of the adhesive removal task **(B, C)**, ladder rung walking task **(D, E)**, and rotarod test **(F, G)**. **(C, E, G)** Changes in the behavioral outcomes of the same cohort at different time points. N = 8–9, ^&&&^ *p* < 0.001 vs Sham; ^*^ *p* < 0.05, ^**^ *p* < 0.01, ^***^ *p* < 0.001 vs ET-1; ^#^ *p* < 0.05, ^##^ *p* < 0.01, ^###^ *p* < 0.001 vs ET-1 + STIM. Data are represented as the mean ± SEM. For two-group comparisons, the continuous variables were analyzed using an independent samples *t*-test at each time point **(A, B)**. For comparisons among three or four groups, continuous variables were analyzed using two-way repeated measures ANOVA followed by Tukey’s *post hoc* tests **(A–C)**. ET-1 indicates endothelin-1; LFP, local field potential; MNG, morroniside; and STIM, stimulation.

As shown in Figure 1, we utilized a protocol that initiates electrical stimulation for 2 weeks starting 14 days after ET-1 administration, followed by a standalone MNG treatment for a continuous 7 days post-stimulation, to explore the impact of combination therapy on recovery. Sensory−motor functions were examined using an adhesive removal task, rotarod test, and ladder rung walking task. Rats undergoing ischemia showed poor performance in all three behavior tests (*p <* 0.001, Figures 1B–G). Compared to postoperative day 1, the ET-1 group on day 35 did not exhibit significant improvement in the adhesive removal task or the ladder rung walking task (Figures 1C, E). However, there was a notable enhancement in latency to stay on the rotarod test for the rats (*p <* 0.001, Figure 1G), seemingly indicating some degree of recovery in muscle strength and endurance except for their sensory function and fine motor skills, 35 days after stroke. At the same time, compared with those of the ET-1 group, rats in the ET-1 + STIM + MNG group exhibited dramatic improvements in sensory−motor functions, with faster sensitivity to adhesive tape removal (*p <* 0.001, Figure 1B), with a longer staying latency on the rotating rods (*p <* 0.001, Figure 1D) and with fewer foot faults of the forelimb (*p <* 0.001, Figure 1F). Additionally, although DBS-alone-treated rats showed few improvements in all three behavior tests, the performance of the ET-1 + STIM + MNG group was significantly better than the ET-1 + STIM group: the sensitivity to the removal of adhesive tape in the adhesive removal test increased 81.67%, the latency of staying on the rotating rods increased 60.83% (*p <* 0.001), and the percentage of foot faults decreased 45.95% (*p <* 0.05). Collectively, our data suggest that the combined therapy can enhance fine motor functions of the impaired forelimb and enhance overall sensorimotor functions, offering greater benefits for functional recovery after ischemic stroke.

**FIGURE 2:**
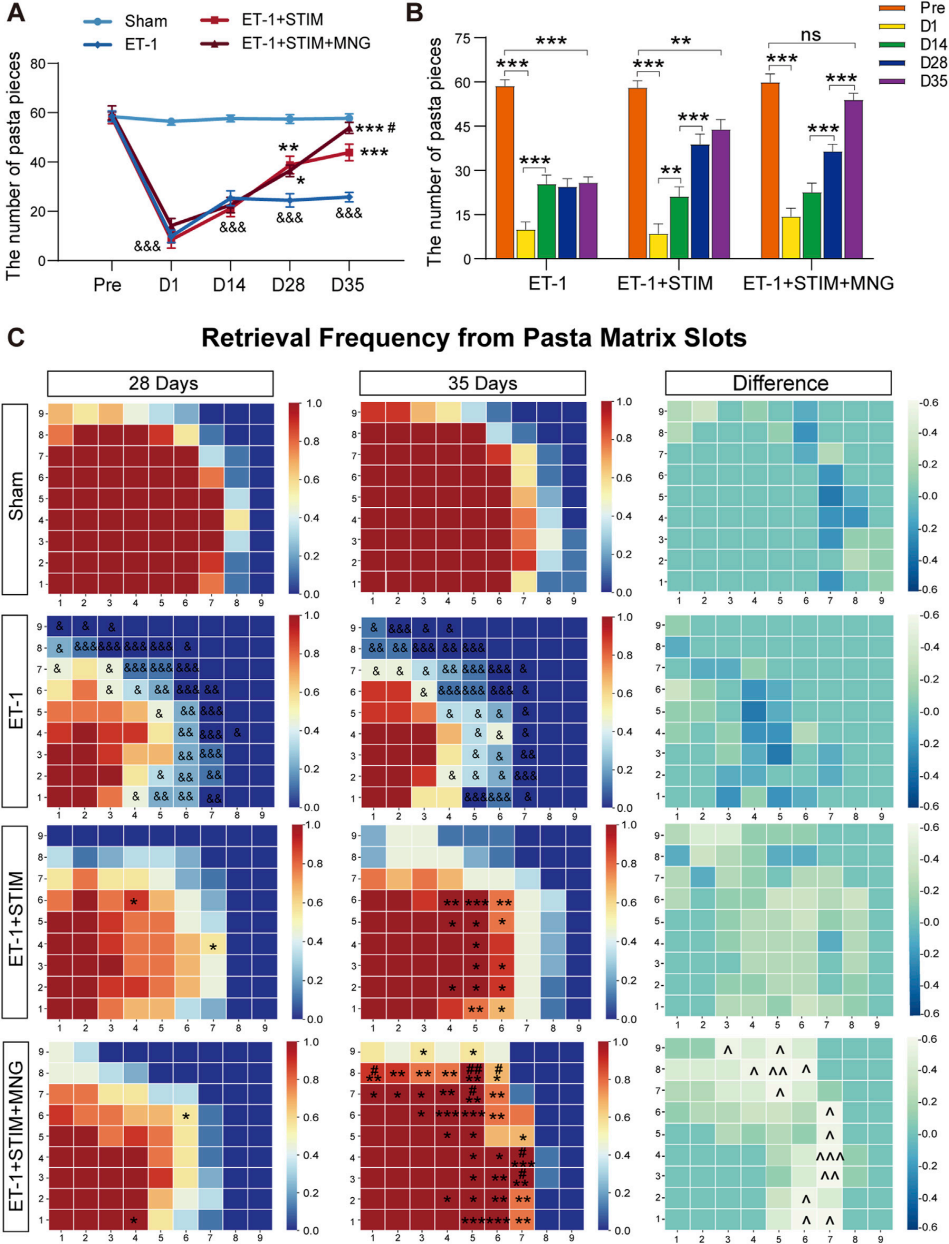
Deep brain stimulation combined with morroniside treatment improves learned skilled fine motor behavior after stroke **(A)** Quantification of the left forelimb reaching performance on the pasta matrix reaching task between cohorts. **(B)** The histogram represents changes in retrieving the number of pasta pieces of the same cohort at different times. **(C)** Graphic depiction of the frequency of pasta retrieval at each matrix position. Matrices were mapped onto the superimposed template, with the left side mirrored to the right side of the template. In each diagram, the bottom left corner corresponds to the locations on the vertical slot where the rats' paws reached the anterior and lateral matrix regions. The color-coding bar stands for pasta acquisition frequency in every position. N = 9, ^&^ *p* < 0.05, ^&&^ *p* < 0.01, ^&&&^ *p* < 0.001 vs Sham; ^*^ *p* < 0.05, ^**^ *p* < 0.01, ^***^ *p* < 0.001 vs ET-1; ^#^ *p* < 0.05, ^##^ *p* < 0.01, ^###^ *p* < 0.001 vs ET-1 + STIM. ^^^ *p* < 0.05, ^^^ *p* < 0.01, ^^^ *p* < 0.01 on day 35 vs day 28. Data are shown as mean ± SEM. Statistical analysis was conducted using two-way repeated measures ANOVA with subsequent Tukey’s *post hoc* tests.

To further assess fine motor function, we tested the reaching ability of the rats’ forelimbs at varying times through performing the pasta matrix reaching task (Figure 2). Therefore, in the initial 24 h following ischemic stroke, rats receiving ET-1 injections exhibited poor reaching capability of the forelimbs relative to their own pre-stroke values and Sham group (*p <* 0.001, Figures 2A, B), indicating the effectiveness of unilateral use of ET-1 to induce ischemia. As expected, a partial spontaneous recovery was shown in the ET-1 group in the absence of any intervention progressively over time. Notably, rats in the ET-1 + STIM and ET-1 + STIM + MNG groups made more successful reach attempts at 35 days post-stroke relative to the ET-1 group (*p <* 0.001, Figure 2A). In particular, rats from the ET-1 + STIM + MNG group experienced a faster recovery, showing a significant improvement of 22.78% over the ET-1 + STIM group (*p <* 0.05, Figure 2A). This indicates that the combination therapy contributed to restoring fine motor control. Figure 2C illustrates the spatial distribution of pasta retrieval. All groups, except the Sham group, displayed obvious impairments in the pasta matrix task after stroke (*p <* 0.05). The spatial distribution patterns varied slightly between the ET-1 and ET-1 + STIM groups over the 2-week treatment period. Animals treated with STIM + MNG outperformed those in the ET-1 and ET-1 + STIM groups, particularly in retrieving pasta pieces from anterior and lateral matrix regions, indicating a spatially specific improvement.

### 2.2 Deep brain stimulation combined with morroniside treatment increased post-infract neuronal activity in stroke rats

**FIGURE 3:**
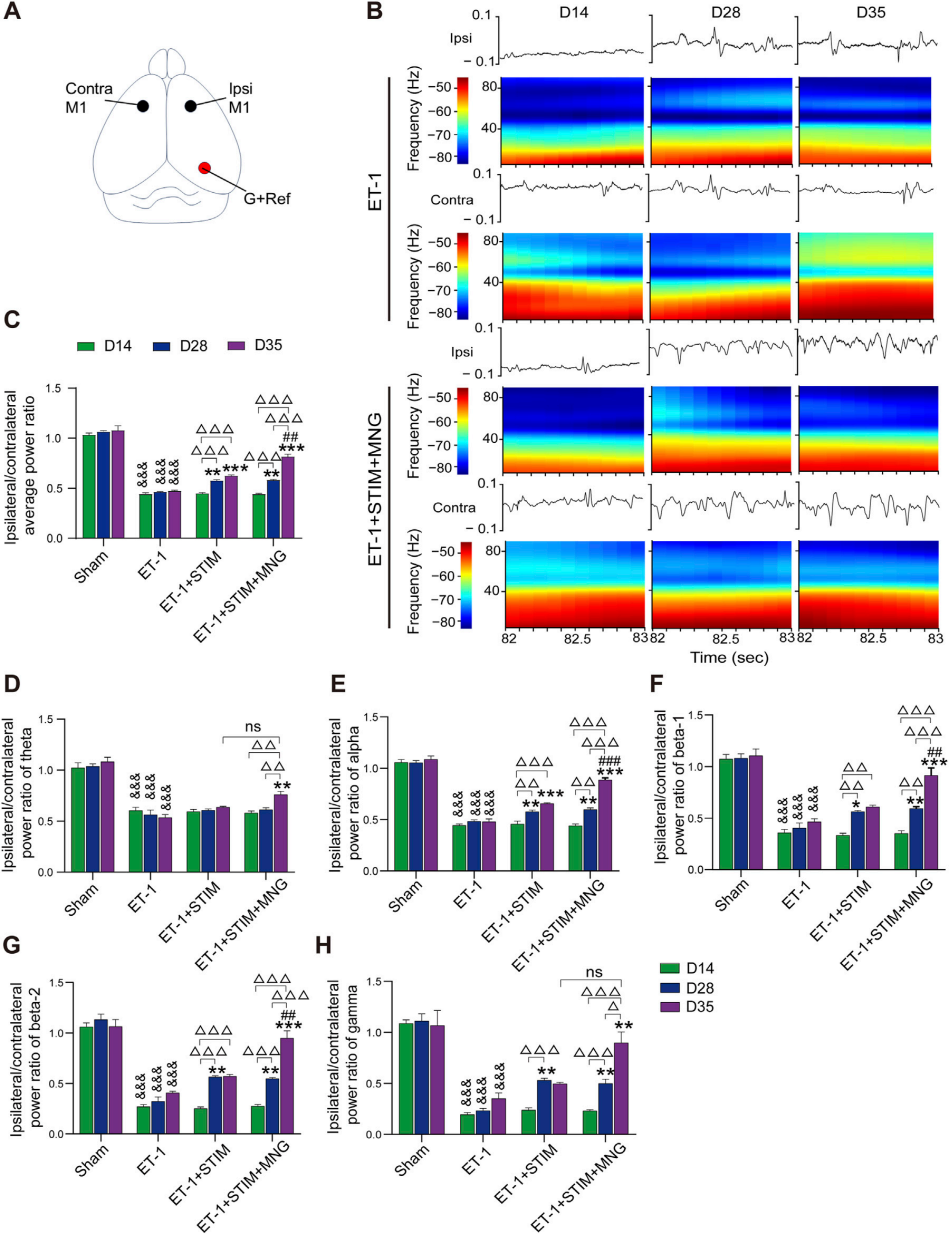
Deep brain stimulation combined with morroniside treatment increased perilesional neuronal activity after stroke. **(A)** Drawing of a rat brain showing the electrode locations for LFP recordings. The black circles represent the location of the recording electrode. The red circle represents the position of the ground wire and the reference electrode. **(B)** Recordings of neuronal activity in local field potential (LFP) responses from the ipsilateral and contralateral hemispheres. Representative images of evoked LFP recordings of the ET-1 and ET-1 + STIM + MNG groups show the changes in the waveform in the second and corresponding spectrogram analysis. *Y*-axis: frequency (Hz); *X*-axis: start of the window; color scales: spectrogram (dB/Hz). **(C)** The histogram represents the quantitative analyses of the average LFP response, and the result is expressed by the overall ipsilesional-to-contralesional averaged power ratio. **(D–H)** The impact of combination therapy on brain oscillation in theta oscillation **(D)**, alpha oscillation **(E)**, beta-1 oscillation **(F)**, beta-2 oscillation **(G)**, and gamma oscillation **(H)**. N = 5, ^&&&^ *p* < 0.001 vs Sham; ^*^ *p* < 0.05, ^**^ *p* < 0.01, ^***^ *p* < 0.001 vs ET-1; ^##^ *p* < 0.01, ^###^ *p* < 0.001 vs ET-1 + STIM; ^Δ^ *p* < 0.05, ^ΔΔ^ *p* < 0.01, ^ΔΔΔ^ *p* < 0.001, n.s., non-significant. Data are represented as mean ± SEM, analyzed using two-way repeated measures ANOVA with Tukey’s *post hoc* tests. Contra indicates contralateral; G, ground; Ipsi, ipsilateral; M1, primary motor cortex; ns, not significant; and Ref, reference electrode.

We ascertained whether alterations in neuronal activity occurred following DBS combined with MNG treatment in stroke rats by performing local field potential (LFP) recordings in the M1 (Figure 3). Fourier transformation was applied to the recorded forelimb-stimulation-evoked LFP responses, yielding the average power in both the ipsilateral and contralateral M1 hemispheres. Figure 3B shows the evoked LFP responses within the ipsilateral (ET-1-injected) and contralateral hemispheres. Considering the impact of contralateral M1 neuronal activity on ipsilateral M1, we analyzed the ipsilesional-to-contralesional power ratio and conducted two-way repeated measures ANOVA tests on the average values. Our results show that there was no lateralization (a difference in both hemispheres) of the spectral power in evoked activity recordings in the Sham group. As expected, rats subjected to stroke showed a remarkable decline in the average ipsilesional-to-contralesional power ratio relative to the Sham group, but no obvious changes were observed at all time points (*p <* 0.001, Figure 3C). Nevertheless, at day 35, the ET-1 + STIM and ET-1 + STIM + MNG groups reversed the declined average spectral power and significantly increased the average ipsilesional-to-contralesional power ratio, relative to that of the ET-1 group (*p <* 0.001, Figure 3C). Noteworthily, the average ipsilesional-to-contralesional power ratio in the ET-1 + STIM + MNG group was 30.37% higher than the ET-1 + STIM group at 35 days (*p <* 0.01, Figure 3C), indicating that combined therapy reduced stroke-induced silencing in the ipsilesional hemisphere and enhanced neuronal activity in the motor system network.

According to a previous report, we divided LFP recordings into five oscillation frequency ranges: theta from 3 to 10 Hz, alpha from 8 to 14 Hz, beta-1 from 15 to 20 Hz, beta-2 from 20 to 35 Hz, and gamma from 35 to 90 Hz (Lake et al., 2017). As a result, we found that the ET-1 group showed significantly lower power values in all five oscillation bands than the Sham group from 14 days to 35 days (all *p <* 0.001, Figures 3D–H). Comparison with the ET-1 group showed that the ET-1 + STIM group only significantly increased the alpha power value (*p <* 0.001, Figure 3E), and that the ET-1 + STIM + MNG group remarkably strengthened all five oscillation power values including the theta (*p <* 0.01, Figure 3D), alpha (*p <* 0.001, Figure 3E), beta-1 (*p <* 0.001, Figure 3F), beta-2 (*p <* 0.001, Figure 3G), and gamma (*p <* 0.01, Figure 3H). Moreover, we found further significant differences in the alpha (*p <* 0.001), beta-1 (*p <* 0.01), and beta-2 (*p <* 0.01) power values in the ET-1 + STIM + MNG group, which remarkedly increased by 35.04%, 49.98%, and 66.29%, respectively, relative to that of the ET-1 + STIM group.

### 2.3 Deep brain stimulation combined with morroniside treatment ameliorated pathological damage to brain tissue in stroke rats

**FIGURE 4:**
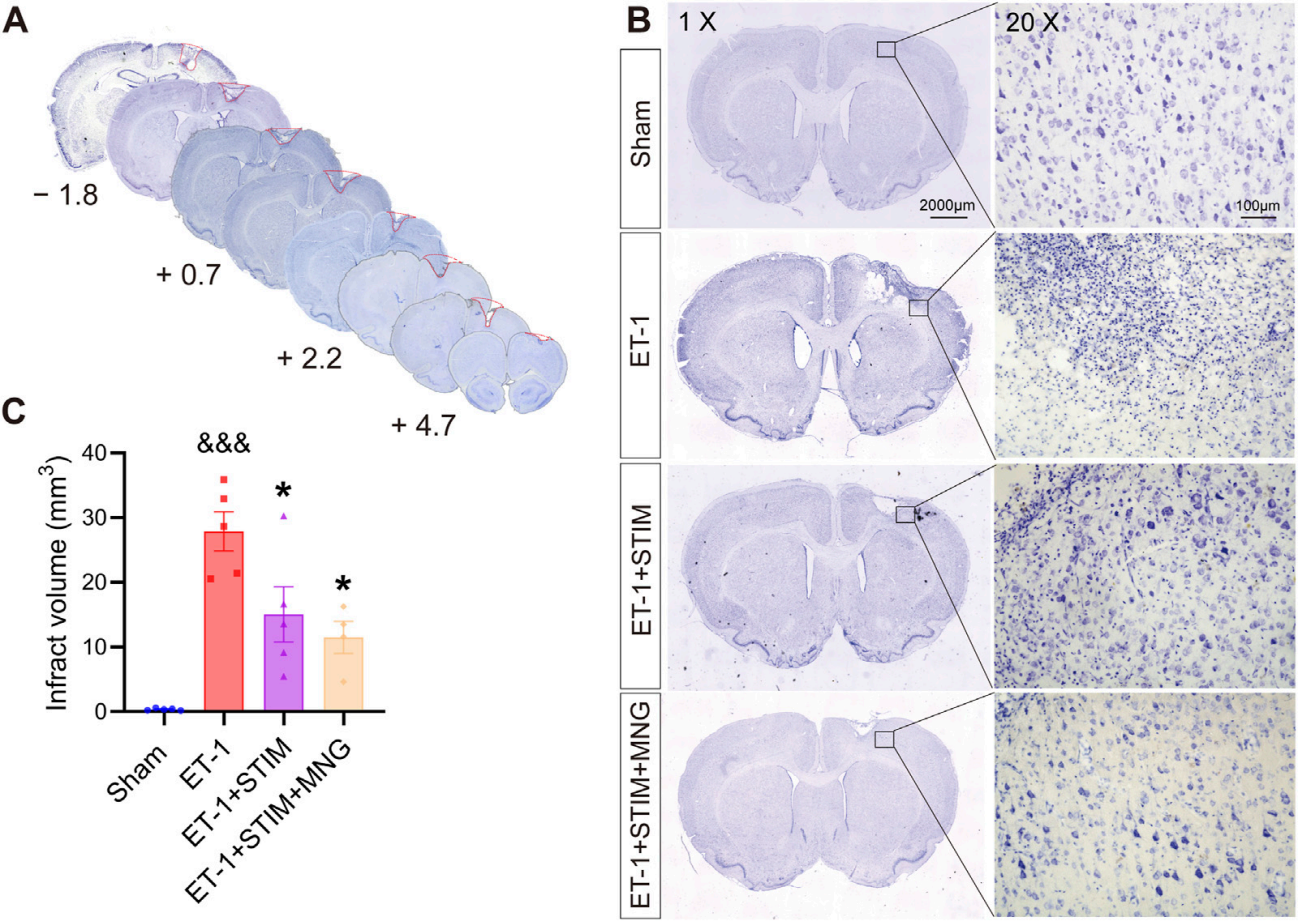
Deep brain stimulation combined with morroniside treatment reduced infarct volume after stroke. **(A)** Infarct volume measurements were taken from 4.7 mm anterior to 1.8 mm posterior to bregma. **(B)** Representative images of Nissl-stained coronal sections of rat brain at 35 days post-stroke. Scale bars, 2000 μm; zoom scale bars, 100 µm. **(C)** The histogram displays the quantitative analyses of infarct volume. N = 4–5, ^&&&^ *p* < 0.001 vs Sham; ^*^ *p* < 0.05, ^**^ *p* < 0.01 vs ET-1 group. Data are expressed as mean ± SEM (one-way ANOVA with Scheffe’s *post hoc* tests).

We conducted Nissl staining to detect the infarct area (Figure 4). As seen in Figure 4A, neurons in the brain tissue of the Sham group exhibited abundant and regular morphologies, and their nuclei were round and clear with deep blue staining of Nissl bodies. Neurons in brain tissue experienced severe damage after ischemic stroke, with an increased number of pyknotic and fragmented neurons, deformed and atrophied cells, and broken and irregular nuclei; meanwhile, the Nissl bodies showed signs of dissolution and necrosis. However, the ET-1 + STIM + MNG group displayed the pyramidal cells with more Nissl bodies stained in the cytoplasm. In addition, we observed that the ET-1 + STIM (14.95 ± 4.30 mm^3^) and ET-1 + STIM + MNG (11.49 ± 2.47 mm^3^) groups showed significantly reduced infarct volume, by 46.56% and 58.93%, separately (*p <* 0.05, *p <* 0.01, Figure 4C), relative to that of the ET-1 group (27.98 ± 3.08 mm^3^).

### 2.4 Deep brain stimulation combined with morroniside treatment enhanced neural stem cell proliferation and migration in the subventricular zone in stroke rats

**FIGURE 5:**
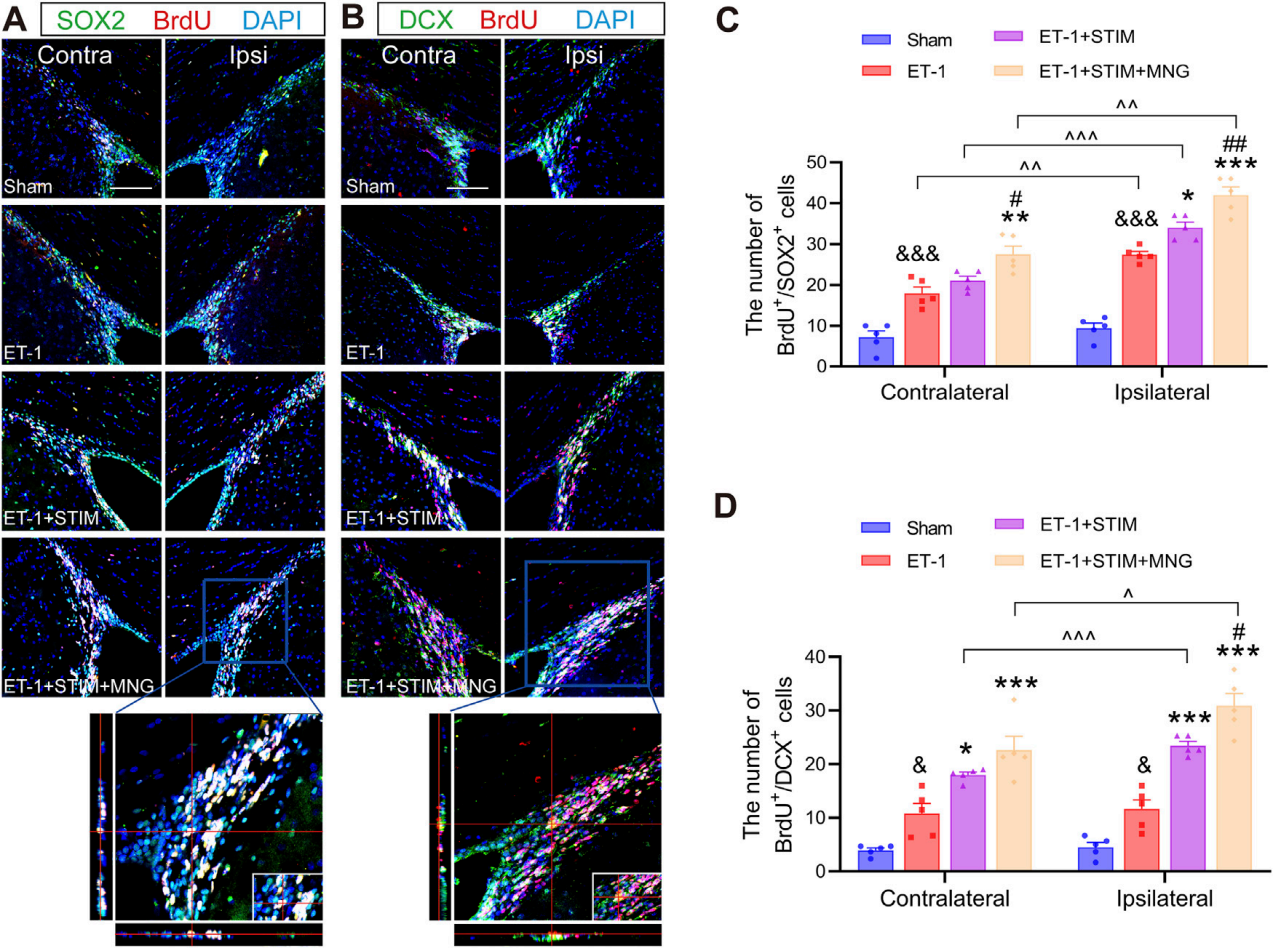
Deep brain stimulation combined with morroniside treatment enhanced neural stem cell proliferation and migration in the SVZ after stroke. **(A)** Representative immunofluorescent show BrdU (red) and SOX2 (green) with the DAPI counterstaining (blue) in both the contralateral and ipsilateral SVZs at 35 days after stroke. The orthogonal view validated the co-localization of BrdU, SOX2, and DAPI in the typical ET-1 + STIM + MNG group. **(B)** Representative immunofluorescent staining of BrdU (red) and DCX (green) with the DAPI counterstaining (blue) in both the contralateral and ipsilateral SVZs at 35 days after stroke. The orthogonal view confirmed the colocalization of BrdU, DCX, and DAPI in the typical ET-1 + STIM + MNG group. Scale bars = 100 µm **(C, D)** Quantitative analyses show the counts of BrdU/SOX2-positive cells **(C)** and BrdU/DCX-positive cells **(D)**. N = 5, ^&^ *p* < 0.05, ^&&&^ *p* < 0.001 vs Sham; ^*^ *p* < 0.05, ^**^ *p* < 0.01, ^***^ *p* < 0.001 vs ET-1; ^#^ *p* < 0.05, ^##^ *p* < 0.01 vs ET-1 + STIM; ^^^ *p* < 0.05, ^^^ *p* < 0.01, ^^^ *p* < 0.01. Results are expressed as mean ± SEM. The comparisons among the four groups were conducted using one-way analysis of variance, followed by Tukey’s *post hoc* tests. The comparisons between the contralateral and ipsilateral SVZs were analyzed using a paired *t*-test. BrdU indicates 5-bromo-2′-dexoyuridine; DAPI, 4,6-diamidino-2-phenylinidole; DCX, Doublecortin; SOX2, SRY-box 2.

Brain sections were co-stained for BrdU/SOX2 (the recognized phenotypic marker for NSCs) and BrdU/DCX (the neuroblast marker) in the SVZ at 35 days after stroke to identify the proliferating and migrating cells, respectively (Figure 5). We found that stroke surgery led to a significantly higher count of BrdU^+^/SOX2^+^ cells on the ipsilateral side when compare with the Sham group (*p <* 0.001, Figure 5C). In addition, a similar trend was also shown in the contralateral SVZ in the ET-1 group (*p <* 0.001). There were more BrdU^+^/SOX2^+^ cells in the ipsilateral side of the ET-1 group than in the contralateral side, indicating that NSC proliferation was induced in the condition of ischemic stroke (*p* < 0.01). Furthermore, relative to the ET-1 and ET-1 + STIM groups, the ET-1 + STIM + MNG group exhibited a notable elevation in BrdU^+^/SOX2^+^ cells in the contralateral SVZ (*p <* 0.01, *p <* 0.05), with an even more substantial rise on the ipsilateral side (*p <* 0.001, *p <* 0.01). The ipsilateral BrdU^+^/SOX2^+^ cell count in the ET-1 + STIM + MNG group was 23.27% higher than that in the contralateral SVZ (*p <* 0.01). Simultaneously, a significant enhancement of BrdU^+^/SOX2^+^ cells was found in the ipsilateral SVZ of the ET-1 + STIM group relative to the contralateral side and the ET-1 group (*p <* 0.05), but no differences were observed in the contralateral side between the ET-1 and ET-1 + STIM groups. These findings suggest that combination therapy further promoted ischemia-induced NSC proliferation, especially in ipsilateral NSCs.

Next, we noted an obvious elevation in BrdU^+^/DCX^+^ cells on the ipsilateral side in the ET-1 group compared to the Sham group, with the increase in contralateral BrdU^+^/DCX^+^ cells similar to that on the ipsilateral side (*p <* 0.05, *p <* 0.05, Figure 5D), suggesting that only a small fraction of stroke-derived NSCs developed into neuroblasts. Additionally, there was a dramatic increase in BrdU^+^/DCX^+^ cells in the ipsilateral SVZ of both the ET-1 + STIM and ET-1 + STIM + MNG groups compared to the contralateral side (*p <* 0.001, *p <* 0.05) and to the ET-1 group (*p <* 0.001, *p <* 0.001). Strikingly, the amount of BrdU^+^/DCX^+^ cells in the ipsilateral side of the ET-1 + STIM + MNG group was 31.53% higher when compared with that observed in the ET-1 + STIM group (*p <* 0.05), but there existed no difference in contralateral SVZ.

Taken together, stroke injury stimulated NSC proliferation and small-scale migration. DBS combined with MNG treatment further enhanced NSC proliferation and migration on the ipsilateral side.

### 2.5 Deep brain stimulation combined with morroniside treatment increased newborn neurons and affected functional neuron differentiation in the ischemic penumbra in stroke rats

**FIGURE 6:**
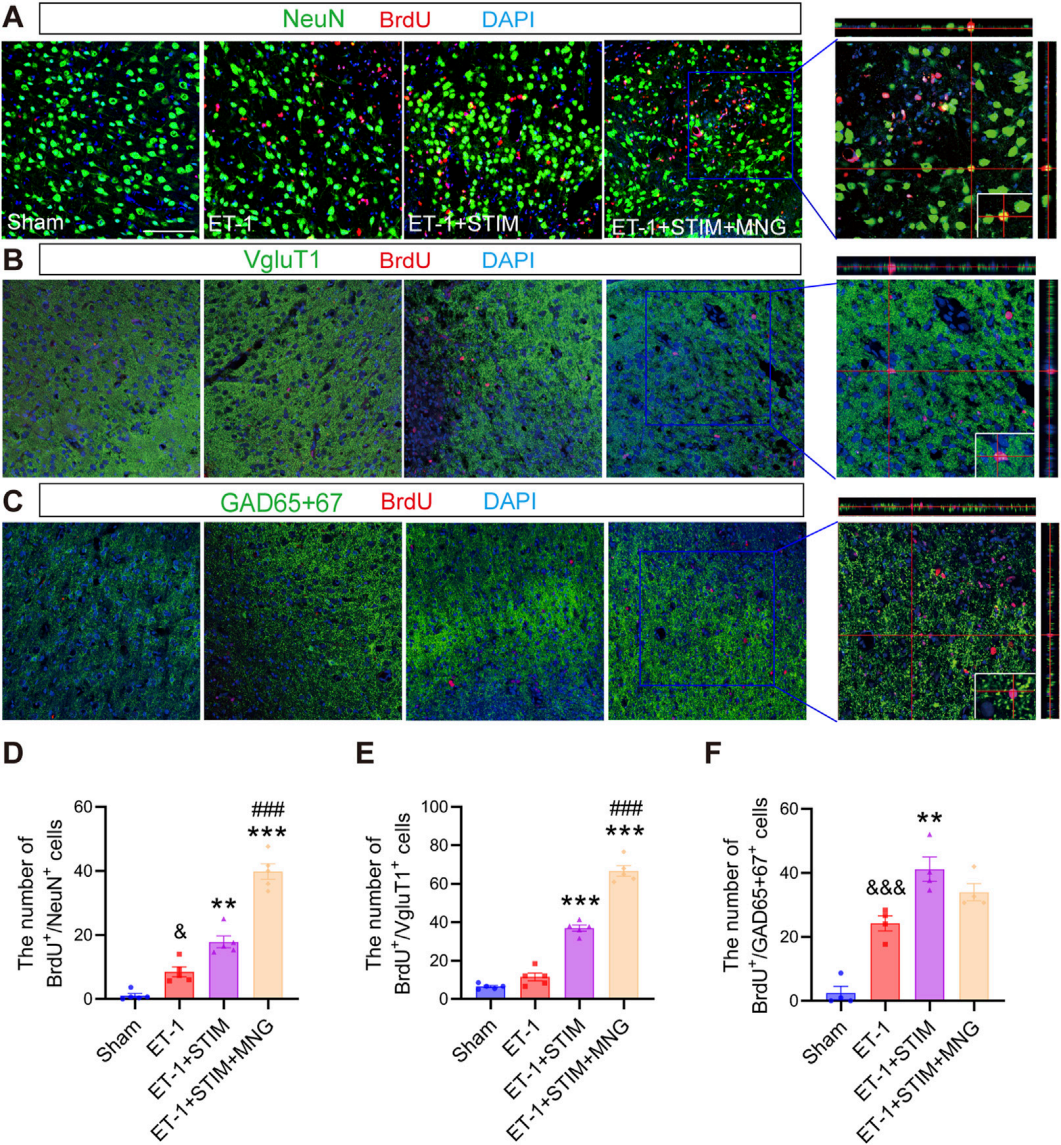
Deep brain stimulation combined with morroniside treatment increased newborn neurons in the peri-infarct zone following a stroke. **(A–C)** Representative triple-labeled images of BrdU (red), NeuN (green), and DAPI (blue) in **(A)**; BrdU (red), VgluT1 (green), and DAPI (blue) in **(B)**; and BrdU (red), GAD65 + 67 (green), and DAPI (blue) in **(C)** at 35 days after stroke. The orthogonal view in a typical ET-1 + STIM + MNG group is positioned in the right panels, representing the co-localization of the three colors. Scale bars = 100 μm **(D–F)** Quantitative analysis of BrdU/NeuN-positive cells **(D)**, BrdU/VgluT1-positive cells **(E)**, and BrdU/GAD65 + 67-positive cells **(F)**. N = 4–5, ^&&^ *p* < 0.01, ^&&&^ *p* < 0.01 vs Sham; ^**^ *p* < 0.01, ^***^ *p* < 0.001 vs ET-1; ^##^ *p* < 0.01, ^###^ *p* < 0.001 vs ET-1 + STIM. Data are mean ± SEM. Statistical analysis was conducted using one-way ANOVA, followed by Tukey’s *post hoc* tests. GAD65 + 67 indicates glutamic acid decarboxylase 65 + 67; NeuN, neuronal nuclei; and VgluT1, vesicular Glutamate Transporter 1.

To verify if NSCs in rats treated with DBS and MNG differentiated into mature new neurons in the penumbra region post-stroke, this study performed co-staining for NeuN/BrdU on sections taken 35 days after stroke induced by ET-1 (Figure 6). As a result, in agreement with the SVZ findings, we observed a higher number of mature BrdU/NeuN-labeled newly proliferated neurons in the penumbra zone in the ipsilateral side of the ET-1 group than in the Sham group (*p <* 0.05, Figure 6D). Likewise, when compared with the ET-1 group (*p* < 0.01, *p* < 0.001), both the ET-1 + STIM and ET-1 + STIM + MNG groups exhibited an apparent elevation in newborn neurons. It was noted that the number of newly formed neurons around the infarct in the ET-1 + STIM + MNG group was 1.23 times higher than that in the ET-1 + STIM group (*p <* 0.001). These findings suggested that combination therapy could more effectively enhance neurogenesis and facilitate NSC differentiation into mature neurons in the peri-infarct brain region.

To identify the effect of combination therapy on neuron types, confocal microscopy was conducted to assess VGluT1 (glutamatergic marker) and GAD65 + 67 (GABAergic marker) expression. As shown in Figure 6, we found significantly more BrdU/GAD65 + 67-positive cells around the peri-infarct zone in the ET-1 group than in the Sham group (*p <* 0.001, *p <* 0.001, Figures 6E, F), but BrdU/VGluT1-positive cells between the two groups have no obvious change. Relative to the ET-1 group, the ET-1 + STIM group demonstrated a significant increase in both types of cells (*p <* 0.001, *p <* 0.01). Notably, the ET-1 + STIM + MNG group exhibited an even more pronounced effect on the proliferation of BrdU/VGluT1-positive cells, with a nearly 6-fold increase in BrdU/VGluT1-positive cells compared with the ET-1 group and a nearly 2-fold increase compared with the ET-1 + STIM group (*p <* 0.001 vs ET-1, *p <* 0.001 vs ET-1 + STIM). These results suggest that combination therapy selectively affects glutamatergic perilesional neurogenesis.

### 2.6 Deep brain stimulation combined with morroniside treatment promoted neuroplasticity in stroke rats

**FIGURE 7:**
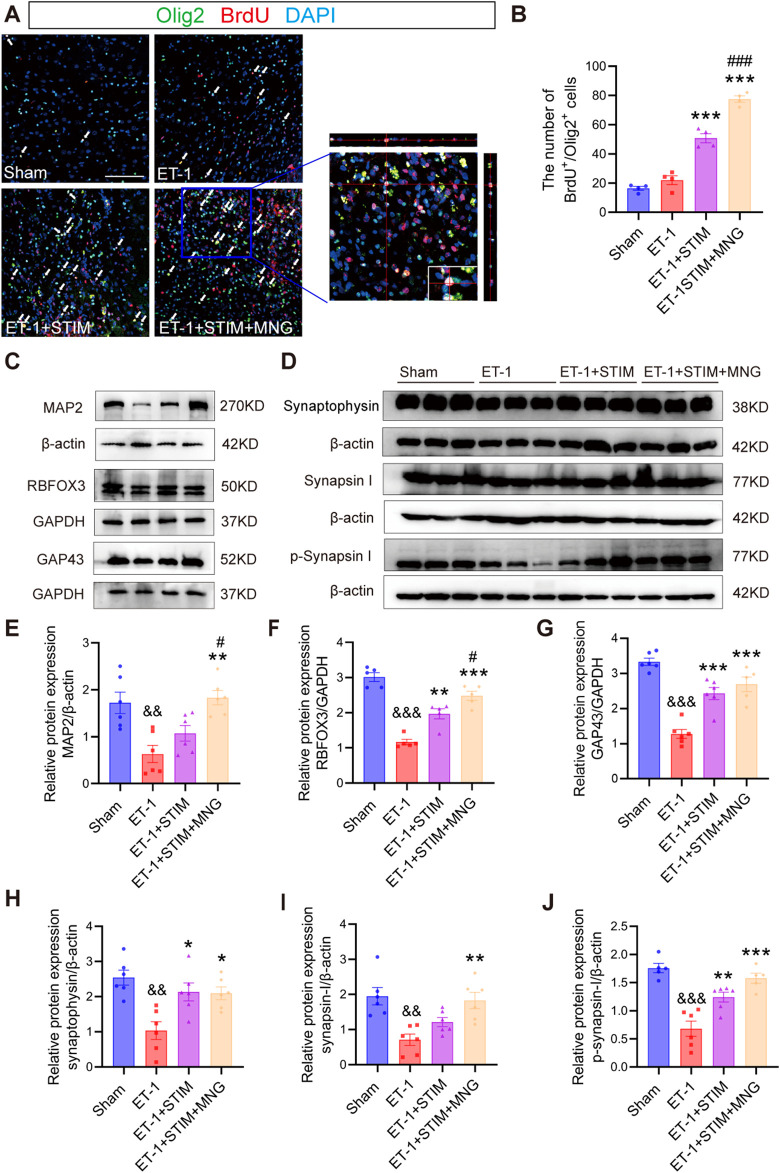
Deep brain stimulation combined with morroniside treatment increased axonal plasticity in the ipsilesional cortex **(A, B)** Representative images and quantitation of BrdU (red)-/Olig2 (green)-positive cells in the peri-infarct area at 35 days after stroke. Orthogonal view verified the co-localization of BrdU, Olig2, and DAPI. Scale bars = 100 μm. **(C)** Representative Western blot images of MAP2, RBFOX3, and GAP43 in the ipsilesional cortex at 35 days post-stroke. **(D)** Western blot images of synaptophysin, synapsin I, and p-synapsin I in the ipsilesional cortex at 35 days post-stroke **(E–J)** Quantitative analyses of MAP2 expression **(E)**, RBFOX3 expression **(F)**, GAP43 expression **(G)**, synaptophysin **(H)**, synapsin I **(I)**, and p-synapsin I **(J)**. N = 5–6, ^&&^ *p* < 0.01, ^&&&^ *p* < 0.01 vs Sham; ^*^ *p* < 0.05, ^**^ *p* < 0.01, ^***^ *p* < 0.001 vs ET-1; ^#^ *p* < 0.05, ^###^ *p* < 0.01 vs ET-1 + STIM. Values are presented as mean ± SEM. One-way ANOVA was used for statistical analysis with Tukey’s and Scheffe’s *post hoc* tests. GAPDH indicates glyceraldehyde-3-phosphate dehydrogenase; GAP43, growth associated protein 43; MAP2, microtubule associated protein 2, and Olig2, oligodendrocyte lineage transcription factor 2.

To investigate whether the combined therapy of DBS and MNG affected oligodendrocyte differentiation in the peri-infarct area following stroke, double-staining of BrdU and Olig2 was carried out in brain sections (Figures 7A, B). According to the results, we found that the number of BrdU/Olig2 positive cells in ET-1 group was not evidently different from that in Sham group at 35 days after stroke, but ET-1 + STIM group significantly promoted the proliferation of Olig2 labeled oligodendrocytes (*p <* 0.001 vs ET-1, Figure 7B). Similarly, the ET-1 + STIM + MNG group also significantly increased BrdU/Olig2 labeled positive cells compared with ET-1 group (*p <* 0.001), and increased by 52.71% compared with ET-1 + STIM group (*p <* 0.001). Thus, our results indicate that combined therapy can further promote oligodendrogenesis after stroke.

In the next experiment, we detected GAP-43, MAP-2, and RBFOX3 levels using Western blot analysis to determine whether newly generated oligodendrocytes affected axonal regeneration (Figures 7C, E, F). We observed a remarkable reduction in MAP2, RBFOX3, and GAP43 levels in the ET-1 group (all *p <* 0.01, Figures 7E–G), suggesting a severe axonal injury occurred after stroke. Although the administration of LCN DBS resulted in only a little increase in the level of MAP2 expression, it significantly elevated RBFOX3 and GAP43 levels relative to the ET-1 group (*p <* 0.01, Figure 7F; *p <* 0.01; Figure 7G). Intriguingly, the ET-1 + STIM + MNG group not only notably elevated the expressions of RBFOX3 and GAP43 (*p <* 0.001, Figure 7F; *p <* 0.001; Figure 7G), but also significantly elevated MAP2 expression levels (*p <* 0.01, Figure 7E) relative to those in the ET-1 group. Importantly, when compared with the ET-1 + STIM group, the levels of MAP2 and RBFOX3 were further elevated by 70.65% and 26.08% in the ET-1 + STIM + MNG group, respectively (*p <* 0.05, Figure 7E; *p <* 0.05; Figure 7F).

In order to assess the effect of DBS combined with MNG treatment on synapses, the expressions of synaptophysin, synapsin I, and p-synapsin I were analyzed (Figures 7G–J). Western blot analysis indicated that all three proteins were expressed much lower in the ET-1 group compared with those in the Sham group (all *p <* 0.01, Figures 7H–J), indicating that synaptic activity was impaired by stroke. We observed a remarkable upregulation the expression of synaptophysin and p-synapsin I in the ET-1 + STIM group (*p <* 0.05, Figure 7H; *p <* 0.01; Figure 7J), accompanied by a trend towards elevated synapsin I levels compared to the ET-1 group. However, we saw no difference in synapsin I expression between the ET-1 and ET-1 + STIM groups. Meanwhile, compared with the ET-1 group, we found greater synaptophysin, synapsin I, and p-synapsin I levels in the ET-1 + STIM + MNG group, which were increased by nearly 3-fold (*p <* 0.05, Figure 7H; *p <* 0.01; Figure 7I; *p <* 0.001; Figure 7J). Furthermore, we observed a trend towards increased expression of synapsin I and p-synapsin I between the ET-1 + STIM and ET-1 + STIM + MNG groups. These results suggest that combined therapy may promote axonal regeneration and synaptogenesis post-ischemic stroke.

## 3 Discussion

Developing new therapeutic strategies to restore post-stroke neurological function in stroke survivors is an urgent priority. Our present work shows that combination of DBS and MNG initiated at 2 weeks post-ischemic stroke in the adult rats not only markedly accelerated functional recovery in both skilled fine motor and non-skilled behaviors, but also enhanced neurogenesis and facilitated the remodeling of brain structure after stroke. These findings indicate the potential of DBS coupled with MNG treatment as a strategy to enhance functional restoration and prolong the window for regenerative repair in ischemic stroke.

Neurogenesis in the adult brain has been found across various species, including humans (Huttner et al., 2014; Ohira et al., 2010; Zhang and Chopp, 2015). Cells expressing markers of newborn neurons have been identified in the ischemic penumbra regions surrounding cerebral cortical infarcts in human brain biopsies (Huttner et al., 2014; Jin et al., 2006). Moreover, animal studies and human neuroimaging data have offered evidence suggesting that endogenous neurogenesis may be linked to the recovery of neurological function (Chen et al., 2010; Koh and Park, 2017; Raber et al., 2004; Zhang and Chopp, 2009). A recent study confirmed synaptic integration of SVZ-derived neurons post-stroke using recombinant rabies virus-based monosynaptic tracing, and silencing these neurons specifically disrupts the formation of synaptic connections and impairs functional recovery (Liang et al., 2019). In addition, our previous work, along with that of others, identified that DBS could modulate neurogenesis and improve motor recovery in rats subjected to stroke (Morimoto et al., 2011; Chan et al., 2018; Wu et al., 2020). In present work, we corroborates these findings by demonstrating that, in comparison with either ET-1-induced stroke or DBS treatment alone, the combination of DBS with MNG significantly augments the pool of neural progenitor cells and the number of immature neurons in the SVZ post-stroke. This is evidenced by an increase in cell counts following double staining with BrdU/SOX2 and BrdU/DCX. Glutamatergic neurons and GABAergic interneurons affect excitatory and inhibitory synaptic transmission, which has an important effect on neuronal excitability and cortical plasticity (Mayor and Tymianski, 2018). Recent researches emphasized that encouraging the SVZ-derived progeny toward ischemic penumbra and differentiation into mature neurons and glutamatergic neurons, could obviously improve functional recovery following stroke. It also demonstrated the reversal of laser stimulation-induced behavioral gains in stroke mice by inhibiting cell proliferation and migration (Song et al., 2017; Liang et al., 2019). Utilizing co-staining methods involving BrdU/NeuN, BrdU/VgluT1, and BrdU/GAD65 + 67, combined therapy was found to boost the differentiation of both new neurons and glutamatergic neurons in the ischemic penumbra, echoing previous findings (Morimoto et al., 2011; Cho et al., 2016; Chan et al., 2018). The parallel occurrence of newly generated neurons and glutamatergic neurons may imply a potential connection between the two. One notable study leveraging immunogold electron microscopy and patch-clamp recordings, presented compelling evidence of NPCs differentiating primarily into glutamatergic neurons, with most of differentiated NeuN and/or neurofilament expressing VgluT1, confirming their glutamatergic identity and function (Yu et al., 2019). In addition, newborn neurons may also facilitate cellular replacement under brain ischemic conditions (Gao et al., 2024; Kanemura et al., 2024; Zhang et al., 2024). For neurons to form functional connections and neural networks, the specific neuron types, such as glutamatergic neurons, is paramount. These process involve that the recovery of functional networks often correlates with increased cortical excitability, where glutamatergic neurons likely contribute to neuronal connectivity and facilitate recovery (Páscoa and Vershure, 2022; Saraswathy et al., 2024). Besides increasing the count of newborn neurons, we also observed that combination therapy could reduce the infarct volume, which may reflect the process of brain remodeling (Shabani et al., 2022). Our data support the notion that DBS combined with MNG treatment strengthens neurogenesis following a stroke, thereby extending the neurorepair window.

Stroke can lead to the loss of oligodendrocytes and their associated myelin, thereby compromising axonal function and leading to white matter dysfunction (Itoh et al., 2015; Shen et al., 2022). However, evidences indicated that increased endogenous oligodendrogenesis supports brain repair processes and alleviates neurological deficits (Zong et al., 2022; Cheng et al., 2024). Olig2 is a marker for immature oligodendrocytes and critical for myelin sheath formation (Zhang et al., 2023). In the present study, while both treatment patterns enhenced oligodendrocyte proliferation, combined therapeutic approach demonstrated a greater effect than DBS alone, suggesting the combined strategy has a more beneficial impact on inducing oligodendrogenesis after stroke. Perilesional expression of GAP43, MAP2 and RBFOX3, key proteins associated with axonal regeneration, was also analyzed. GAP43 is associated with neurite extension and is the inherent determining factor for axonal growth, regeneration, and synaptic plasticity (Carmichael et al., 2006; Juan et al., 2014). The findings of the current study revealed a concomitant upregulation of all three proteins in rats treated with a combination of DBS and MNG. This observation aligns with a previous finding that upregulating the expression of GAP43 and MAP2 through a Nogo-A inhibitor, ameliorate myelin damage and promote regeneration (Zhao et al., 2021). Moreover, in transgenic mice, overexpression of GAP43 has been observed to induce the spontaneous formation of new synapses and enhanced neuronal sprouting (Aigner et al., 1995). Our results suggest that the combined treatment is at least indirectly involved in axonal regeneration. Regrettably, we did not directly observe the progression of axonal regeneration. Utilizing biotinylated dextran amine to label sprouting axons and track the direction and extent of axonal growth, or employing electron microscopy to visualize morphological changes during axonal regeneration, will be helpful in further elucidating the mechanisms of the combined treatment. In addition to its role in axonal regeneration, combined treatment also promoted synaptogenesis. Synaptophysin is a major presynaptic vesicle protein that is critical for synaptogenesis and synaptic remodeling (Xia et al., 2017), while synapsin I is integral to neurotransmitter release (Bolay et al., 2002). Recent evidences have shown that LCN DBS increased the expression of perilesional synaptophysin, a crucial marker for long-term potentiation, in parallel with functional recovery, indicating a potential improvement in synaptic plasticity and post-ischemia motor recovery attributable to DBS (Machado et al., 2013; Cooperrider et al., 2014). We demonstrated that rats treated with a combination of DBS and MNG increased synaptophysin, synapsin I, and p-synapsin I expressions in the peri-infarct tissue 35 days post-stroke compared with untreated rats. Collectively, our study indicates that combined treatment contributed to neural plasticity.

Building on the observed enhancements in neural plasticity and brain remodeling by combination treatment, we further examined its role in functional recovery. In individuals suffering from stroke and in rodent models, LFP is diminished following a stroke, suggesting a reduction in synchronous neuronal activity within brain oscillations (Ramanathan et al., 2018; Bönstrup et al., 2019). The obtained data are in consistence with previous studies and further demonstrate that combination treatment increased neuronal excitability and reduced lateralization between brain hemispheres, as revealed by changes in LFP recordings. In particular, these neurophysiological changes were concomitant with the improvement in skilled reaching ability on the pasta matrix reaching task and of sensory−motor functions on other behavioral tests. In humans and animal models, measurement of cortical activity evoked by sensory stimulation reveals reduced neuronal excitability in the peri-infarct cortex as well as in connected cortical regions on both the ipsilesional and contralesional sides. This diminished inter-regional signaling is a direct measure of lost connectivity. This is also evidence of the initial loss of responsiveness of neurons that react to stimulation of the forelimb following a stroke. Nevertheless, enhancing excitatory signaling in the tissue adjacent to the infarct promotes recovery (Clarkson et al., 2010; Lim et al., 2014; Lake et al., 2017; McDonnell and Stinear, 2017; Sequeira et al., 2020; Joy and Carmichael, 2021). Our findings suggest that increased neuronal excitability from combination therapy may contribute to better manifestations of motor control and greater sensorimotor function recovery. We underscore the parallel surge in neuronal activity, functional rejuvenation, and significant physiological alterations, including neuroplasticity and remodeling. These findings suggest the need for further studies to establish a definitive causal linkage between neurogenesis and recovery (Liang et al., 2019). Furthermore, we focused on changes in different oscillations after stroke since alterations in brain oscillation have been previously linked to recovery outcomes after stroke. In a long-term study, evidence has suggested that groups of function and behavior were closely related to the presence of each oscillation band (Ballermann et al., 2001). Notably, we observed that the combination treatment effect was the most pronounced in beta oscillations, which was related to motor function and was considered to make a vital impact on movement impairment (Adam et al., 2015; Rabiller et al., 2015). Several studies investigated that stroke disrupts beta oscillations; however, higher beta oscillations are related to improved motor function. Furthermore, normal beta oscillations were supposed to be the basis of maintaining activity in the motor cortex (Rabiller et al., 2015; Thibaut et al., 2017; Leonardi et al., 2022). Consistent with previous evidence, our results show that beta-1 and beta-2 oscillations were decreased at all time points in stroke rats and, more importantly, combination therapy restored beta-1 and beta-2 oscillations to values showing no obvious difference from those observed in the Sham group. Particularly, the increased beta oscillations were accompanied by a higher speed of motor improvement in all behavioral tests in combination-therapy-treated rats and higher values of gross average power. This result demonstrates that combination therapy improves cortical excitability in stroke rats by restoring beta oscillations, resulting in functional recovery and suggesting that beta oscillations may be applied to be a physiological biomarker of motor recovery in stroke.

**FIGURE 8:**
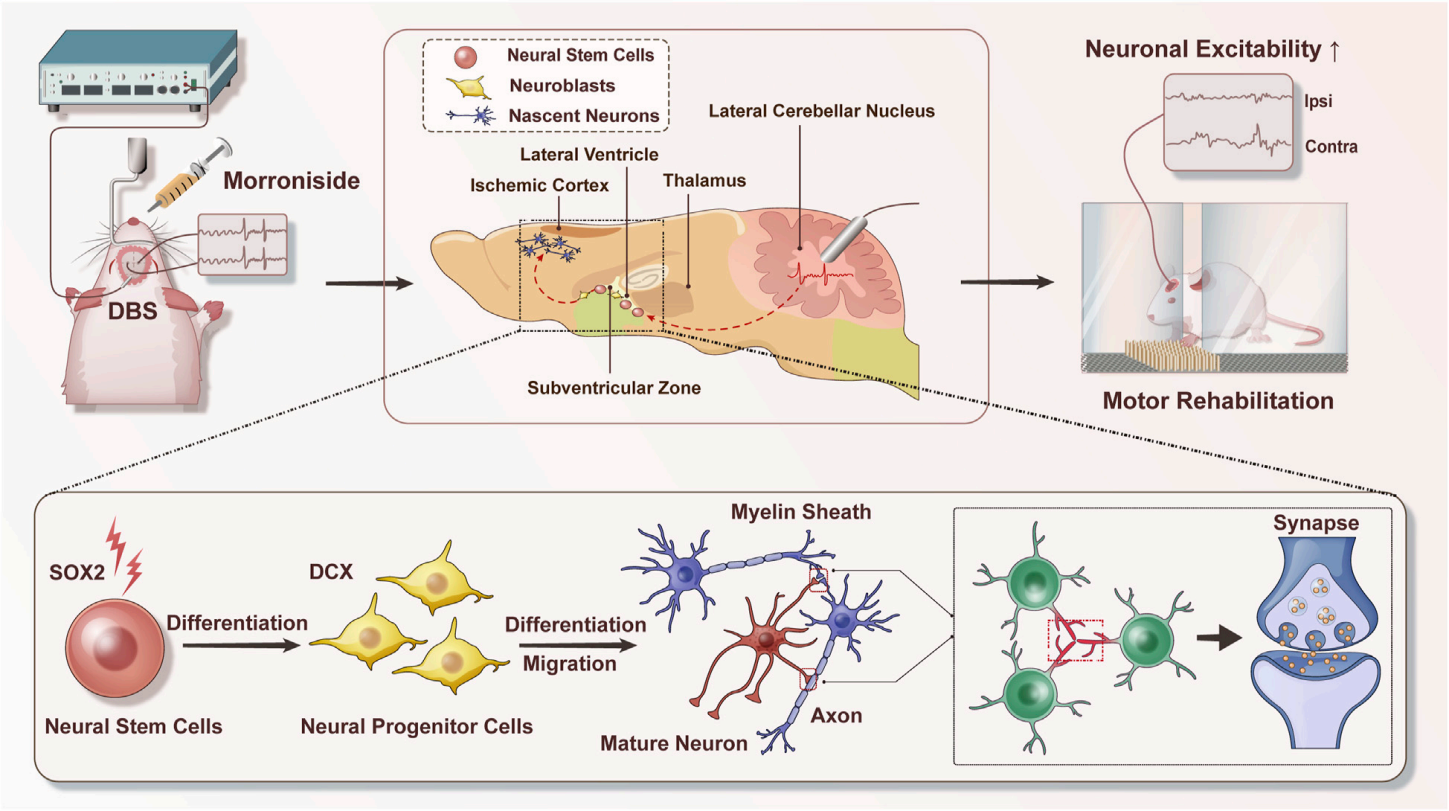
Schematic diagram of deep brain stimulation combined with morroniside in the treatment of post-stroke motor disorders. Combination therapy could promote neurogenesis and facilitate the remodeling of brain structure; moreover, the proposed therapy could enhance neuronal excitability and restore sensory−motor dysfunction. During this procession, endogenous neural stem cells are activated to differentiate into neuroblasts, which subsequently migrate to the ischemic injury area and differentiate into various types of neurons. Axonal connections and synaptic transmission between neurons could also be enhanced. These changes are ultimately beneficial for stroke recovery. DBS indicates deep brain stimulation.

**FIGURE 9:**
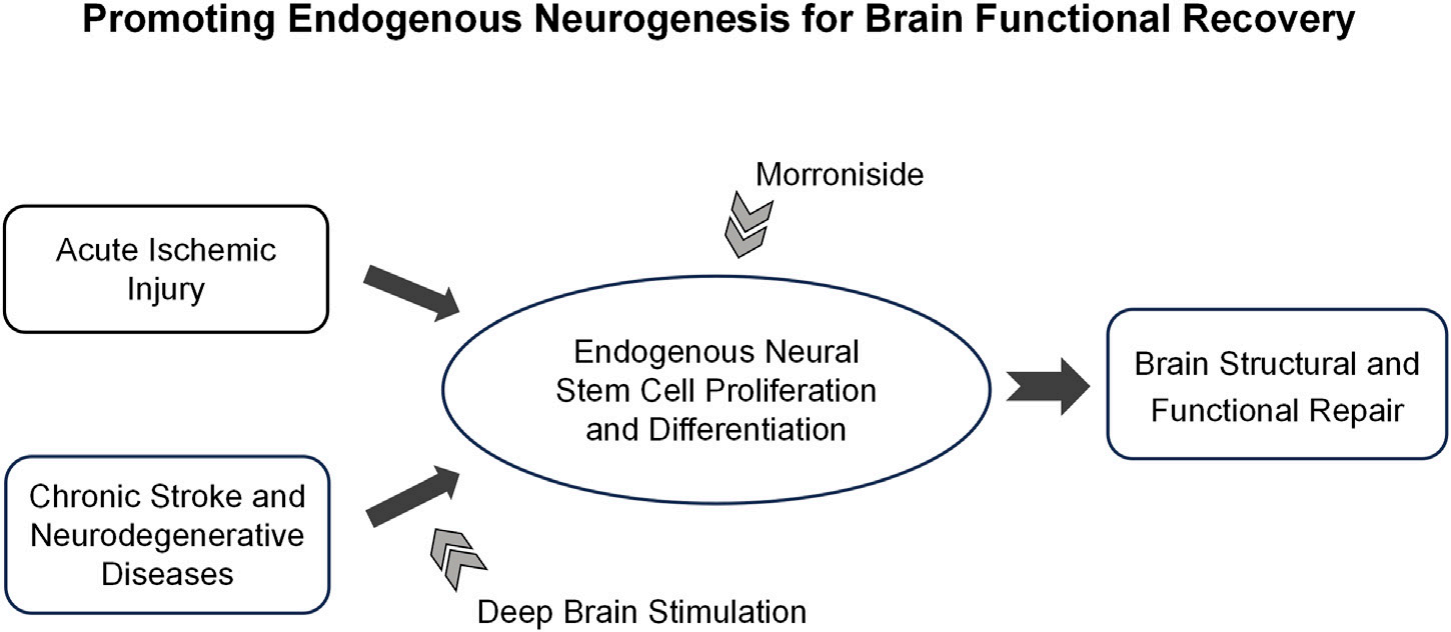
The hypothesis of deep brain stimulation combined with morroniside treatment on neurological disorders. The diagram presents a novel strategy for promoting brain repair through personalized treatment, which is tailored according to the progression and characteristics of neurological diseases. In the acute phase of stroke, morroniside treatment could promote stroke-induced neurogenesis and diminish cerebral damage. In conditions such as chronic stroke and certain neurodegenerative diseases like Parkinson’s and Alzheimer’s, endogenous neurogenesis is significantly limited. The application of deep brain stimulation in combination with morroniside treatment could further augment the capacity for neurogenesis, thereby facilitating brain remodeling. This promising approach holds potential for achieving functional restoration in these chronic conditions.

Taken together, our results present novel evidence that the combination of DBS and MNG treatment could may serve as an effective therapeutic approach for the robust enhancement of endogenous neurogenesis and facilitating the remodeling of brain structure. Moreover, the proposed treatment could restore neuronal excitability and enhance functional recovery, highlighting the potential of this approach to extend the therapeutic time window of MNG (Figure 8). Accordingly, we propose a new strategy to augment the capacity of endogenous neurogenesis as a therapeutic approach for chronic stroke and certain neurodegenerative diseases, including Parkinson’s disease, in which endogenous neurogenesis is markedly limited. Cerebral ischemia could trigger a transient and weak neurogenic response in the acute phase of stroke. Morroniside treatment could augment neurogenesis, thereby facilitating brain regeneration and repair while mitigating cerebral damage. In chronic stroke and certain neurodegenerative diseases, the capacity for brain neurogenesis is limited. Neurogenesis can be activated through the application of DBS, and this effect can be further augmented by combining it with pharmacotherapy to enhance brain remodeling, thereby leading to functional recovery (Figure 9).

This study has some limitations that should be considered. Firstly, the major limitation of our work is the lack of long-term follow-up. This absence of extended tracking might obscure a complete portrayal of long-term recovery outcomes. This deficiency motivated our future endeavors to address the gap. Secondly, although our immunofluorescence analyses hint at a role for delayed DBS coupled with morroniside in post-stroke neurogenesis, possibly via enhancing functional recovery, we acknowledge that other mechanisms may also contribute, e. g., the neuroprotective effect or the recruitment of available neurons. Our subsequent studies will focus on elucidating the specific role of endogenous neurogenesis in mediating functional gains. Thirdly, our study observed a beneficial effect of the combination treatment on neurogenesis and recovery, yet the intricate regulatory pathways within neural networks remain elusive. Future studies will involve virus-mediated synaptic tracing to map neuronal connections and unravel the underlying molecular mechanisms, thus strengthening our understanding of the treatment impact. Moreover, in the context of clinical applicability, it may be advantageous to explore the timing for delayed initiation of therapy to establish treatment paradigms, thereby offering tailored therapeutic strategies for patients at differing stages of disease progression.

## 4 Methods

### 4.1 Preparation of drug

Following the previous description, the extraction of purified morroniside was performed from the sarcocarp of *C. Officinalis* purchased from Tong-Ren-Tang (Beijing, China). Based on high-performance liquid chromatography, the final purity reached 98.5% (Sun et al., 2014b).

### 4.2 Animals

A total of 60 adult male Sprague-Dawley rats (10-week-old, from Beijing Vital River Experimental Animal Co. Of China) were used, of which were assigned to either a Sham group (n = 12) or an ET-1-injection group (n = 48). Several rats were excluded due to criteria non-compliance related to grasping ability in pasta matrix training before stroke (Sham rats: 1, Experimental rats: 4) and 11 were excluded due to ET-1-induced ischemic surgery fatalities (n = 9) or excessive injury severity (n = 2). The remaining 33 rats were grouped into ET-1 (n = 11), ET-1 + STIM (n = 11), and ET-1 + STIM + MNG (n = 11) groups. The experimental procedures were approved by the Animal Care and Use Committee of Xuanwu Hospital, affiliated with Capital Medical University, under Ethics Approval No. XW-20210417-02, in compliance with the 2017 regulations on laboratory animals set forth by the Ministry of Science and Technology. The rats were housed under specific-pathogen-free (SPF) conditions, maintained at 25 °C with a 12-h light/dark cycle.

### 4.3 Behavioral tests

#### 4.3.1 Pasta matrix reaching task

The pasta matrix reaching task was extensively developed to examine fine motor control, and rats are instructed to reach, retrieve, and grasp the pasta matrix via one small shift prior to eating this pasta matrix (Ballermann et al., 2001). The rats underwent a 2-week pretraining period on the pasta matrix reaching task, according to previous description, practicing three times daily at 8 a.m., 12 p.m., and 7 p.m. (Cooperrider et al., 2014; Wu et al., 2020). In each behavioral testing period, food provisions were controlled to about 14.68 g/day. In the process of training, pasta was presented on the right matrix sides to establish a dominant forepaw in rats. Subsequently, the rats were put into a cage that had one vertical slot in its front panel for the rat’s forelimbs to reach, break, and eliminate the pasta pieces from half of the pasta matrix (9 × 9 pieces) on the side opposite to the dominant limb. The last 3 days of the second week’s performance included pre-stroke baseline and pre-stroke pasta matrix reaching performance data. Rats who were outside two standard error of the means from the mean performance were withdrawn. In this longitudinal study, each rat received a pasta matrix task test for 30 min, and the pasta retrieval number and distribution were recorded.

#### 4.3.2 Ladder rung walking task

As described previously, the ladder rung walking task was conducted to rate the forelimb paw slips/misplacements for the sake of evaluating motor agility and coordination (Metz and Whishaw, 2009). Before the surgery, the rats underwent a 7-day training protocol. Each rat was restricted to crossing the horizontal ladder where rungs were irregularly arranged (100 cm long, 8 cm wide, and 40 cm above ground) in an identical direction that ended at the goal box. During the testing, the number of missteps was video-recorded, and the average of three measurements for each rat was determined. Any slight or deep paw slips and complete misses were deemed to be errors. Data are represented by the ratio of errors to total steps.

#### 4.3.3 Adhesive removal task

Sensorimotor function was evaluated using an adhesive removal test, which was carried out as previously described with some adjustments (Sun et al., 2020). Pre-surgery training was carried out in each rat three times per day for three consecutive days. Briefly, this study utilized one piece of round adhesive-backed tape (100 mm^2^) as an ipsilateral tactile stimulus, which was attached to the palmar surface of the ipsilateral forepaw. The last-day performance was averaged to establish a pre-stroke baseline and used as the pre-stroke adhesive removal performance data. Rats that were outside of two standard error of the means from the mean performance were excluded from subsequent studies. During the testing, the time it took for each rat with the purpose of removing the corresponding stimulus (tape) from the involved forelimb was recorded. Each rat completed three trials lasting a maximum time of 3 min, with inter-trial intervals of 5 min. The data are represented by the average of three measurements.

#### 4.3.4 Rotarod test

The rotarod test was adopted for evaluating motor coordination and muscle endurance in line with previous description with some modifications (Zeng et al., 2018). At first, the rats were habituated to a stationary rod for 2 min. Subsequently, a fatigue rotator (Institute of Materia Medica Chinese Academy of Medical Science, China) was gradually accelerated to 20 r/min and each rat was trained for 5 consecutive days to adapt to the rod rotator. Only rats that were able to stay on a rotating rod for 170–180 s at a constant speed of 20 r/min were included in the experiment. Data obtained 1 day preoperatively were recorded as baseline data. The length of time each rat stayed on the rotating rod was recorded. Three trials were measured with an interval of more than 10 min. The results are expressed as the average of three measurements.

### 4.4 Endothelin-1-induced ischemia model

After 2 weeks of training, each rat was anesthetized with sodium pentobarbital (2% sodium pentobarbital dissolved in sterile saline, 75 mg/kg). Afterwards, the rats were fixed onto stereotaxic frames (David Kopf, Los Angeles, CA, United States), the calvaria was exposed with a midline incision, and three burr holes were drilled corresponding to the motor cortex contralateral to the left paw for endothelin-1 (ET-1, Millipore, Burlington, MA, United States) injection. In addition, the coordinates for each 800 pmol ET-1 injection, diluted to 2 μL in relation to bregma, were set as follows: for the first point, anterior–posterior (AP) = −1.0 mm, medial–lateral (ML) = −2.5 mm, and dorsoventral (DV) = −2.3 mm; for the second point, AP = +1.0 mm, ML = −2.5 mm, and DV = −2.3 mm; for the third point, AP = +3.0 mm, ML = −2.5 mm, and DV = −2.3 mm (Park et al., 2015). A 28 G needle was used to penetrate the brain, starting the injection after a 1-min delay at a flow of 0.5 μL/min, followed by a 5-min pause prior to needle withdrawal. The rats in the Sham operation group received an identical surgical process, with saline injection rather than ET-1. Postoperatively, the animals were put onto a 37 °C heating plate until they completely recovered from anesthesia. Finally, the rats were returned to their corresponding cages.

### 4.5 Deep brain stimulation surgeries and experiments

After 1 week of free recovery, the rats underwent a second surgery to implant custom-made macro-electrodes in the area of interest. The electrodes included both recording and stimulating electrodes. The stimulating electrodes were made of PFA-insulated tungsten (California Fine Wire, Grover Beach, CA, United States) with a diameter of 175 μm and an exposed tip of 1 mm (resistance less than 1 mΩ) Briefly, the stimulating electrode was implanted in the LCN opposite to the ET-1 injection sites by drilling a burr hole under coordinates relative to bregma: AP at −11 mm, ML at +3.6 mm, and DV at −6.3 mm (Park et al., 2015). Recording electrodes were installed at the same time as the stimulation electrodes in rats, and holes were punched above the rat’s cortex with the dental drill in coordinates associated with bregma as follows: for the first installation set, AP = +1 mm, ML = +2.5 mm, and DV = 0 mm; for the second set, AP = +1 mm, ML = −2.5 mm, and DV = 0 mm to install the recording electrodes; and for the third set, AP = −8 mm, ML = −2 mm, and DV = 0 mm to install a baseline and a ground line (Lake et al., 2017). Electrodes were then immobilized in the exposed skull with dental acrylic. In order to demonstrate the accuracy of the electrode placement, this study triggered reproducible forelimb movements by elevating the stimulation frequency. Rats not exhibiting obvious motor responses were excluded from this study.

Seven days post-surgery, the stimulating electrodes were connected through a cable to a stimulator (Plexon, Dallas, TX, United States). The stimulator was employed to control isochronous stimulation at 30 Hz at 80% of the motor threshold; meanwhile, the pulse width was kept at 70–130 μA and 400 μs The lowest current that induced a visible motor response in the ipsilateral forepaw, torso, or vibrissae was determined as the motor threshold for the LCN stimulation (Wu et al., 2020). The ET-1 + STIM and ET-1 + STIM + MNG groups received a 2-week stimulation for 8 h/day. Although the rats in the Sham and ET-1 groups were connected to the stimulator, no stimulation was applied. After the conclusion of the stimulation procedure, the ET-1 + STIM + MNG group commenced a regimen of morroniside, which was dissolved in normal saline. This prepared solution was then administered intragastrically to the rats at a consistent dose of 270 mg/kg each day, spanning a treatment duration of 7 days. The rats in the Sham, ET-1, and ET-1 + STIM groups were concurrently administered an identical volume of normal saline, ensuring uniformity in treatment across the groups. Meanwhile, all animals were subject to intraperitoneal injections of 5-bromo-2′-dexoyuridine (BrdU, 50 mg/kg body weight, B5002, Sigma-Aldrich, St. Louis, MO, United States) daily for totally 21 days starting after the 14-day period to label newborn cells.

### 4.6 Electrophysiological recordings

Induced LFP recordings were performed on days 14, 28, and 35 after the stroke. After anesthetizing the rats with sodium pentobarbital, the contralesional forelimb of the rats was stimulated once for 10 s, and then the stimulation was repeated 30 times (10 Hz, 2.5 mA, and 3 pulses of current; each pulse stimulation consisted of stimulation for 2 ms with a delay of 32 ms and stimulation for 2 ms with a delay of 64 ms), and the LFP signals of the 30 pulse stimulations were recorded continuously using an OmniPlex (Plexon, Dallas, TX, United States) *in vitro* multichannel recording system (Lake et al., 2017). We analyzed from 3.5 s to 8 s after each stimulus from the 7th stimulus to the 30th stimulus.

### 4.7 Brain sections and protein preparations

After the treatment phase, the rats were sacrificed under anesthesia on day 36. The rats underwent transcardial perfusion first with 0.9% saline followed by 4% phosphate-buffered paraformaldehyde until their limbs stiffened. Afterward, their brains were harvested and post-fixed in 4% phosphate-buffered paraformaldehyde for a day, and then cryoprotected with 30% sucrose (Zhu et al., 2019). Cryoprotected brains were prepared into coronal sections (20 μm). In the preparation of proteins, a portion of rats was perfused using pre-chilled phosphate-buffered saline (PBS) for dissection. Brain tissues from the peri-infarct regions were collected, and Sham controls were also dissected from the ipsilesional cortex. The olfactory bulbs and cerebellum were removed while dividing the brain into hemispheres, followed by dissection of the ipsilesional hemisphere to collect cortical tissue. This involved removing the midbrain, corresponding residual cortex, and subcortical tissue along the ventral limit of the hypothalamus and midbrain, as well as along the line of the corpus callosum, respectively. Collected tissue instantly frozen at −80 °C until further processing. Regarding protein preparation, the brain tissues were homogenized and lysed in pre-chilled RIPA buffer (Applygen, Beijing, China) that contained phosphatase inhibitors (Roche, Basel, Switzerland) to extract proteins (Zheng et al., 2024). Following homogenate centrifugation (140,00× g at 4 °C, 30 min), the supernatants were gathered as the total protein. And then a bicinchoninic acid protein assay kit (Applygen, Beijing, China) was adopted for measuring protein expression levels.

### 4.8 Infarct volume quantification

Every 10th section containing infarcts was put onto the slide in sequence for Nissl staining. A light microscope (Nikon AX/AX R with NSPARC, Tokyo, Japan) was employed to observe the stroke area on each slice, and based on Image Pro-plus software, the area of the lesion was quantified. The “contralateral area” is identified as the right brain region that is located on the side opposite to the induced ischemic lesion. The equation below adopted for calculating the volume (Wu et al., 2020):

$$Stroke\;volume\;\left(\mu m^{3}\right)=\sum_{1-number\;of\;slices}^{20}\left[\left(contralesional\;hemisphere\;area-ipsilesional\;hemisphere\;area\right)\times 20\times 10\right]\;$$

### 4.9 Immunofluorescence staining

For BrdU staining, 0.1 M PBS that included 0.3% Triton X-100 was added for section permeabilization, followed by 26 min of incubation with 2 N HCl under 37 °C prior to blocking with 5% donkey serum (017-000–121, Jackson ImmunoResearch Laboratories, Philadelphia, PA, United States) (Wu et al., 2020). Afterwards, primary antibodies were supplemented to incubate samples under 4 °C, including rat anti-BrdU (1:200, ab6236), rabbit anti-Doublecortin (DCX) (1:200, ab18723), rabbit anti-SRY-box 2 (SOX2) (ab92391), rabbit anti-Neuronal nuclei (NeuN) (ab177487), rabbit anti-Oligodendrocyte lineage transcription factor 2 (Olig2) (ab109186), rabbit anti-Glutamic acid decarboxylase 65 + 67 (GAD65 + 67) (ab183999), or rabbit anti-Vesicular Glutamate Transporter 1 (VgluT1) (ab227805) (1:200, Abcam, Cambridge, MA, United States). Thereafter, primary antibody detection was conducted with suitable Alexa Fluor 488- and Alexa Fluor 594-labeled secondary antibodies (A21206 and A21203, Life Technologies, Carlsbad, CA, United States). We counterstained with 4,6-diamidino-2-phenylinidole (DAPI) (ZSGB-BIO, Beijing, China) to label cell nuclei. Fluorescence signal visualization was completed using a confocal microscope (Nikon AX/AX R with NSPARC, Tokyo, Japan). For each sample, three brain sections were fluorescently labeled, with images captured at ×20 magnification for SOX2 and DCX, and at ×10 magnification for NeuN, VgluT1, GABA, and Olig2. We focused predominantly on the SVZ migration stream region, while cortical neurons were sampled from the ischemic penumbra region. The number of nerve cells in each image was manually counted and then averaged for analysis. Images were analyzed by researchers blinded to treatment.

### 4.10 Western blotting

The proteins were dissolved in 5× sodium dodecyl sulfate-polyacrylamide gel (SDS-PAGE) sample buffer and subsequently heated to 95 °C for 10 min. Then, proteins were separated in an SDS-PAGE gel and electrically transferred onto an anitrocellulose membrane (Zheng et al., 2024). We performed immunodetection using the below primary antibodies: Microtubule associated protein 2 (MAP2) (1:2000, ab32454), Neuronal nuclei (RBFOX3/NeuN) (1:1,000, ab18723), Growth associated protein 43 (GAP43) (1:2000, ab75810), synaptophysin (1:16,000, ab32127), synapsin I (1:1,000, ab64581) (Abcam, Cambridge, MA, United States), phospho-synapsin I (p-synapsin I) (1:1,000, sc12913, Santa Cruz, CA, United States). Then, horseradish peroxidase-labeled anti-rabbit, anti-mouse, or anti-coat secondary antibodies were added for further membrane incubation. Finally, the ECL kit (Biotides, Beijing, China) was utilized for visualizing immunoreactive proteins on the membrane.

### 4.11 Statistical analysis

In this study, all data are indicated to be the mean ± standard error of the mean (mean ± SEM). We used SPSS 26.0 (IB M, NY, United States) for statistical analysis. For assessing significance between two groups, a two-tailed independent samples *t*-test was utilized. In the case of multiple group comparisons, we applied one-way ANOVA or two-way repeated measures ANOVA, complemented by Tukey’s and Scheffe’s tests for multiple comparisons. Data comparison from the contralateral and ipsilateral sides within the same animal employed a two-tailed paired *t*-test. The distribution data for the pasta matrix reaching task were analyzed using the Mann-Whitney U-test. The term ‘N’ indicates the used number of rats. *P <* 0.05 was shown to be of statistical significance.

## 5 Conclusion

Thrombolytic recanalization intervention with a limited therapeutic window is the only option available for small subsets of patients with cerebral ischemia. Targeting regeneration and repair offers a potential alternative for stroke patients, providing a wider treatment window. Nevertheless, there are no reparative approaches for ischemic stroke. In this work, DBS in combination with MNG treatment initiated at 2 weeks post-ischemic stroke in the adult rats displayed diminished infarction size, enhanced neuronal excitability and improved functional impairment after 35 days post-stroke. The underlying mechanism is the promotion of endogenous neurogenesis and brain remodeling. Our study shows that combination therapy of DBS with MNG is a new strategy for patients with chronic stroke and neurodegenerative diseases.

## Data Availability

The raw data supporting the conclusions of this article will be made available by the authors, without undue reservation.
